# Resilience of temperate peatland vegetation communities to wildfire depends upon burn severity and pre‐fire species composition

**DOI:** 10.1002/ece3.9912

**Published:** 2023-04-10

**Authors:** G. Matt Davies, Alan Gray, Simon C. Power, Rut Domènech

**Affiliations:** ^1^ School of Environment and Natural Resources The Ohio State University Columbus Ohio USA; ^2^ UK Centre for Ecology and Hydrology Penicuik Scotland; ^3^ Consortium of Environmental Policies of Terres de l'Ebre (COPATE) Amposta Spain

**Keywords:** *Calluna vulgaris*, Composite Burn Index, heathland, moorland, species composition, species diversity, species richness

## Abstract

Peatland ecosystems are of global conservation and environmental importance storing globally significant amounts of ancient carbon, regulating regional temperatures and hydrological regimes, and supporting unique biodiversity. Livestock grazing, land‐use change, drainage, nutrient and acid deposition, and wildfire threaten the composition and function of many peatlands including those in the uplands of the United Kingdom. Presently, little is known about either the short‐ or long‐term effects of wildfires within these systems in the UK. Our study aimed to evaluate how plant communities respond to wildfires across a range of vegetation communities, soil types, and burn severities. We evaluated wildfire burn severity using the ground‐based Composite Burn Index adapted for treeless peatlands. Using paired burned–unburned plots, we quantified differences in the abundance of plant families and functional groups, vegetation diversity, and community composition. Multivariate differences in composition between burned and unburned areas were used as an index of community resilience to fire. Plots in heathland communities with shallow organic soils burned at the highest severities and had the greatest reductions in plant diversity and richness. There were significant declines in plot‐scale species richness and diversity with increasing burn severity. Graminoids were resilient to fire whilst Ericaceae tended to increase with higher severity. Bryophyte composition was substantially altered—pleurocarpous species declined and acrocarpous species increased with greater burn severity. Community resilience was related to ground layer burn severity with higher burn severity driving greater changes in communities. Wildfire effects on temperate peatlands are a function of fire weather and site environmental and ecological characteristics. Management policy should ensure that the risk of severe wildfires is mitigated to protect ecosystem function and biodiversity. This will require system‐specific fire management prescriptions across the gradient of peatland soil and vegetation types.

## Graphical abstract

Peatland wildfires are expected to be a growing problem under climate change, but we have little knowledge of how temperate systems respond to varying fire severity. We investigated differences in the diversity and composition of paired burned and unburned areas across five wildfires in the United Kingdom that burned at differing severities. Plant community diversity and resilience were significantly associated with both burn severity and community type with most differences occurring among cryptogams.

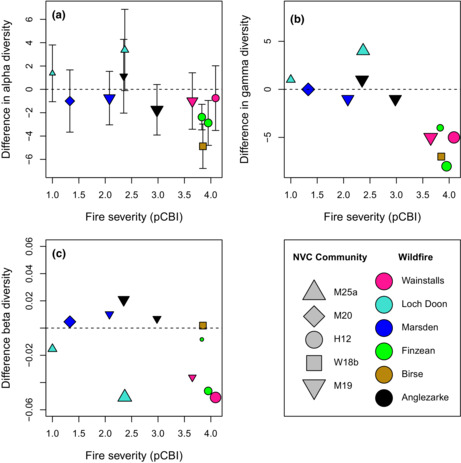

## 1 INTRODUCTION

Peatland ecosystems occur globally but differ significantly in their structure, and abiotic and biotic conditions (Page & Baird, 2016; Xu et al., 2018). Peatlands as a whole are of significant current global conservation and research interest due to the important ecosystem services and functions they perform (Bonn et al., 2016) including, for example, regulation of hydrological regimes and water quality (Brown et al., 2015), the provision of habitat for wildlife (e.g., Newey et al., 2016), opportunities for recreation and hunting (Maltby, 2010), as well as the production of forage for livestock grazing (Palmer, 1997). Notably, peatlands store large below‐ground stocks of ancient carbon that are estimated to be equivalent to a quarter of all global soil carbon and three‐quarters of total atmospheric carbon (Yu, 2011). There is significant concern about the implications of climate change for the preservation of peatland carbon stocks and peatlands' net carbon balance (Ferretto et al., 2019; Gallego‐Sala et al., 2010; Yu et al., 2011). Smouldering peat fires can result in a variety of environmental and human health impacts (Cascio, 2018; Watts & Kobziar, 2013) including substantial instantaneous fluxes of ancient stored carbon to the atmosphere (Conard et al., 2002; Davies et al., 2013; Turetsky & Wieder, 2001) and subsequent changes to soil CO_2_ and CH_4_ fluxes (e.g., Bergner et al., 2004; Gray et al., 2021).

Understanding ecosystem resilience is of critical importance in the context of changing climatic conditions and disturbance regimes. Ecological resilience can be broadly understood as the magnitude and character of changes in ecosystem functions and structures following disturbance (Gunderson, 2000). Increasing wildfire activity on peatlands concerning as repeated events has been shown to reduce peatlands' ecological and hydrological resilience to burning (Kettridge et al., 2015; Sherwood et al., 2013). Current national and international land‐use policies (e.g., UK Government, 2021) place significant emphasis on the conservation and restoration of peatlands to protect and enhance their carbon storage potential (Joosten et al., 2016; Moomaw et al., 2018). Many peatlands, including those in the United Kingdom, have been impacted by a range of disturbances including drainage (Sloan et al., 2018), peat extraction (Alexander et al., 2008), overgrazing (Stevenson & Thompson, 1993), and insensitive use of prescribed burning (Harper et al., 2018). These impacts, in interaction with atmospheric nutrient deposition (e.g., Bragazza et al., 2006; Paal et al., 2010) and the effects of global climate change (Kettridge et al., 2019; Turetsky et al., 2011), are driving significant degradation in many peatland areas.

Predicting the ecological outcomes of fires on peatlands can be complex due to their contingency on the interaction of fire weather, abiotic site characteristics, and pre‐fire community composition. Thus, for example, peatland burn severity has been related to (i) hydrogeological setting and peat moisture content (Hokanson et al., 2016; Lukenbach et al., 2015); (ii) pre‐fire vegetation composition (Davies et al. 2016a); (iii) vegetation fuel structure and moisture (Grau‐Andrés et al., 2017, 2018); and (iv) soil type (Grau‐Andrés et al., 2018; Maltby et al., 1990). Understanding the magnitude of the threat posed by wildfires in temperate peatlands requires a better understanding of their resilience. Whilst it is fairly well‐established that significant gradients can exist in peatland burn severity (e.g., Davies et al. 2016a; Jones et al., 2009; Kolden & Rogan, 2013), previous research has revealed contrasting results with regard to the relationship between burn severity and ecological resilience. Tundra peatlands have, for example, demonstrated relatively rapid recovery of some vegetation components following wildfire (Bret‐Harte et al., 2013), though elsewhere in arctic and subarctic regions, fire has been associated with fundamental changes in permafrost dynamics and site hydrology that can drive shifts in vegetation state from forest to wetland (e.g., Stralberg et al., 2018). There has been rather less research in temperate regions, but the studies by Maltby et al. (1990) and Legg et al. (1992) on British peatlands demonstrate that severe fires can cause long‐lasting changes in environmental conditions and vegetation composition. Similarly, the palaeoecological work by Davies (2016) has suggested that state transitions can occur in relation to fire. Studies based on lower severity‐prescribed burns have demonstrated peatlands to have relatively high ecological resilience to individual fires (Grau‐Andrés et al., 2018; Grau‐Andrés, Davies, et al., 2019; Grau‐Andrés, Gray, et al., 2019).

In the United Kingdom (UK), peatlands are an abundant ecosystem type covering approximately 12% of the total land area (Trenbirth & Dutton, 2019). Blanket peatlands in the UK uplands are widely used for hunting and livestock grazing and many have evidence of human utilization that dates back millennia (e.g., McCarroll et al., 2017; Swindles et al., 2016). Use of traditional managed burning remains a fundamental component of peatland management in the UK and has a long history both there and within botanically‐similar European heathlands (e.g., Davies et al., 2022). Notwithstanding a degree of controversy over the long history and continuing use of managed burning within these ecosystems (Davies et al., 2016b), wildfires are a relatively regular occurrence (Davies & Legg, 2016) and there is concern about their increasing prevalence, particularly in the context of a changing climate (Perry et al., 2022). Currently, there is a lack of evidence concerning the effects of variation in wildfire severity, the ecological impacts of wildfires in general, and the relative resilience of different peatland habitat types.

In this study, we aimed to characterize the range of short‐term resilience responses of peatland plant communities affected by wildfires that burned in the UK uplands in the springs of 2011 and 2012, years noted for the prevalence of large and severe wildfires (Davies et al. 2016a). Following Gunderson (2000), we consider ecosystem resilience to be reflected in the magnitude and character of changes in key ecosystem properties in relation to unburned conditions. Previous research on these fires has demonstrated significant variation in burn severity and fuel consumption as a function of community type and fire weather (Davies et al. 2016a) and alteration of CO_2_ and CH_4_ fluxes (Gray et al., 2021). The overarching goal of the present study was to understand how wildfire‐induced alterations to peatland vegetation differ across burn severity gradients and between distinct plant communities. Our specific objectives were to (i) relate burn severity to changes in vegetation diversity and richness at multiple spatial scales; (ii) evaluate the effects of varying fire severity on the abundance of key plant functional types; and (iii) assess how plant community resilience relates to variation in fire severity.

## 2 METHODS

We monitored vegetation community structure across six different wildfires (Table 1) that burned in the uplands of northern England and Scotland during the springs of 2011 and 2012. Sites were selected from a database of wildfire compiled using information provided by regional Fire and Rescue Services, local landowners, and academic partners. Our six sites were selected to provide fires that exhibited moderate to high fire severities in locations that captured the North–South and West–East gradients of bioclimatic conditions that exist in the British Isles. Two (one in the case of the Birse fire) plots were located within each fire and chosen to represent the range of burn severities visible during site reconnaissance with local stakeholders. Each plot was composed of a burned subplot that was paired with a subplot in an adjacent unburned area. Similar paired‐plot designs have been used in previous studies (e.g., de Groot et al., 2009; Johnstone & Kasischke, 2005; Kasischke et al., 2005). We were careful to ensure that plots were established where we were confident that pre‐fire fuel conditions across the fire line were similar and in regions of the fire line known to have been actively extinguished.

**TABLE 1:** Locations, dates, and environmental conditions associated with the six wildfires surveyed in the study.

Wildfire	Angelzarke	Birse	Finzean	Loch Doon	Marsden	Wainstalls
Location	N England	NE Scotland	NE Scotland	SW Scotland	N England	N England
Lat/Long	53.658° N, 2.569° W	57.007° N, 2.724° W	57.025° N, 2.702° W	55.214° N, 4.393° W	53.596° N, 1.976° W	53.777° N, 1.928° W
Fire Date	29 Apr 2011	23 Mar 2012	30 Mar 2012	29 May 2011	9 Apr 2011	30 Apr 2011
DMC	35	16	30	3	9	35
DC	98	42	62	12	35	118
pCBI	3.09 ± 0.61	3.85 (single obs)	3.93 ± 0.10	1.96 ± 0.83	1.68 ± 0.35	3.80 ± 0.24
pCBI ground	0.92 ± 0.46	1.63 (single obs)	1.57 ± 0.07	0.84 ± 0.38	0.49 ± 0.17	1.61 ± 0.17
NVC Comms.	*Calluna vulgaris – Eriophorum vaginatum* blanket mire (M19)	*Pinus sylvestris* – *Hylocomium splendens* woodland; *Vaccinium myrtillus* – *Vaccinium vitis‐idaea* subcommunity (W18b)	*Calluna vulgaris – Vaccinium myrtillus heath* (H12)	*Molinia caerulea – Potentilla erecta* mire (M25a); Adjacent to sitka spruce (*Picea sitchensis*) plantation	*Calluna vulgaris – Eriophorum vaginatum* blanket mire (M19) *Eriophorum vaginatum* blanket mire (M20)	*Calluna vulgaris – Eriophorum vaginatum* blanket mire (M19) Scattered *Calluna vulgaris – Vaccinium myrtillus* heath (H12)
Soil Type	Histosol	Podzol	Podzol	Histic gleysol	Histosol	Histosol
Deep Peat?	Yes	No	No	No	Yes	Partial
MMaxT (°C)	12.7	12.2	12.2	12.7	12.5	11.8
MMinT (°C)	6.1	3.5	3.5	4.5	5.3	5.1
TAP (mm)	1294	780	780	1721	1028	1024
Drainage?	Yes	No	Possible	Yes	Yes	Limited (windfarm)
Burning?	Yes	No	Yes (+ cutting)	No	Limited	Yes
Erosion?	Yes	No	No	No	Yes (Gullies)	Yes

*Note*: DMC and DC are, respectively, the Duff Moisture Code and Drought Code values from the Canadian Fire Weather Index System for the day of the wildfire (see Davies et al. 2016a for details). pCBI values are reported giving the mean value (±1 standard deviation), and the overall and ground layer scores (see Methods). Soil types and peat depth were derived from UK Soil Observatory Maps with deep peat classified as an O‐layer >50 cm. Mean annual maximum and minimum temperature, and mean total annual precipitation, were determined from UK Met Office data for the closest weather station to the fire. Aerial photographs were examined to determine whether there was visual evidence of drainage ditches, managed burning, or erosion feature (e.g., “peat hags”) in the vicinity of the fire during the last decade.

Vegetation community type and site abiotic conditions varied substantially between our selected wildfires (Table 1). Those in northern England occurred across communities that could generally be classified as mires on deep peat where the dominant species were *Eriphorum* spp. and *Calluna vulgaris* (L.) Hull (hereafter *Calluna*). A number of locations were notably wetter and were more sedge‐dominated with lower cover of *Calluna* and greater cover of *Eriophorum* spp. and *Trichophorum caespitosum* (L.) Hartm. Sites in Scotland covered a more diverse array conditions and included heathlands on shallow organic soils with abundant *Calluna* and *Pteridium aquilinum* (L.) Kuhn, and bogs dominated by *Molinia caerulea* (L.) Moench, *Myrica gale* L., and *Sphagnum* spp. Broad vegetation type (i.e., mire versus heathland) was relatively consistent within fires and specific community types somewhat unique to each. This makes it difficult to explicitly separate site/fire‐level effects from those associated with the vegetation community. We can, however, still reliably examine broad cross‐site gradients in responses to fire.

Burn severity and vegetation data were recorded over the summers of 2011 and 2012, approximately 6–18 months after the fires. Initial scoping visits were used to select plot locations within the burn perimeters that captured a representative range of severities. Burn severity was recorded using the Peatland Composite Burn Index (pCBI) method described in Davies, Domènech et al. (2016) and that was itself based on Key and Benson's (2006) CBI protocol. The Composite Burn Index has previously been used by other authors to estimate burn severity in forested peatland ecosystems (Boby et al., 2010) and has been related to changes in soil microbial communities following tundra/boreal wildfires (Belova et al., 2014). The pCBI method involved the establishment of 20‐m diameter, circular pCBI plots within which semiquantitative fire impact criteria were separately scored for two ecological strata—vegetation (surface pCBI) and soil characteristics (ground pCBI). The final pCBI score is the sum of these values.

We randomly located eight 1 m^2^ quadrats within each subplot where we recorded the presence of all vascular plants and cryptogams. As far as possible plants were recorded to species level. Liverworts specimens (*Cephalozia* spp. and *Cephaloziella* spp.) were generally recorded to genus level. A number of individual plants could not be resolved to species level so soon after fire. For this reason, prior to analysis, we combined records where there was taxonomic uncertainty or inconsistency in the taxonomic level to which species had been identified. This led to us combining all liverworts to the lifeform level, whilst *Sphagnum* spp., *Dryopteris* spp., *Agrostis* spp., and *Cladonia* spp. were grouped to the genus level. Nomenclature follows Stace (2019) for vascular plants and Atherton et al. (2010) and Smith (2004) for bryophytes. Plots were assigned to a standard UK National Vegetation Classification (NVC) plant community based on the composition of the unburned vegetation, following Averis et al. (2004). All data associated with the paper (including plot locations) are available in the Supplemental Information and are archived with OSF (DOI: https://doi.org/10.17605/OSF.IO/R463E).

### 2.1 Statistical analyses

All statistical analyses were completed using R 3.6.1 (R Core Team, 2019). Copies of the data analysis scripts are provided in the Supplemental Information and are archived with OSF (DOI: https://doi.org/10.17605/OSF.IO/R463E).

#### 2.1.1 Burn severity effects on species richness and diversity

The effect of fire severity on species diversity was assessed at multiple spatial scales from quadrat‐level alpha diversity to gamma diversity at the whole fire scale. Differences in plot‐level alpha diversity were assessed as the difference in mean quadrat‐level species richness between burnt and unburnt subplots. Plot‐level gamma diversity differences were defined as the difference in the total number of unique species recorded in each subplot. Beta diversity was assessed at the subplot level using the “betadisper” function from the “vegan” package (Oksanen et al., 2019), and we again examined differences between each burned and unburned subplot pair. Beta diversity thus represents the compositional differences among quadrats in a subplot. All plot‐level diversity/richness differences were evaluated in relation to pCBI using Pearson's correlations (function “cor.test”). Finally, we examined fire‐level effects on diversity by assessing differences in mean richness between all burned and unburned subplots within a fire. Differences in fire‐level gamma diversity were also examined by calculating the total number of species in all burned and unburned subplots in each fire.

#### 2.1.2 Burn severity effects on key plant functional types

Plant functional traits are known to influence species responses to fire (e.g., Schwilk, 2015). In the absence of information on the traits of the vascular and cryptogamic species we encountered, we utilized a number of plant orders, families, and subfamilies as substitutes for plant functional types. We examined differences in the frequency of eight key species groups that included vascular plants (Ericoideae, Poaceae, Cyperaceae, and Vaccinoideae) and bryophytes (Hypnales, Sphagnales, Dicranales, and Jungermanniale). Differences were visualized in relation to plot pCBI (fire severity) and NVC community. Species were assigned to families, subfamilies, orders, or plant functional types based on the number of observations and differences in key traits. For example, Ericoideae and Vaccinoideae both belong to the Ericaceae, but species in the former lack the ability to spread rhizomatously. Likewise, the bryophyte orders used differ substantively in growth form, flammability (moisture content), and typical placement during community successional development.

#### 2.1.3 Plant community resilience in relation to varying burn severity

The resilience of community composition to variation in burn severity was assessed using Non‐Metric Multidimensional Scaling. Species abundance was calculated as the mean frequency across the eight quadrats in the subplot. We utilized the “metaMDS” function with the Bray Curtis dissimilarity matrix and up to 500 iterations. As our data were (mean) frequencies, and we were interested in both the absolute and relative extent of species' recovery as an indicator of resilience, we did not apply any standardization. We examined solutions with two to five dimensions, evaluating the change in stress with a scree plot and selecting a final solution that balanced minimum stress with ease of interpretation (fewer dimensions). All subplots (burned and unburned) were ordinated together, and we quantified resilience by calculating the Euclidean distance between burned and unburned subplot pairs in the ordination. This expresses resilience as the difference in composition between adjacent burned and unburned areas. Greater distance between paired subplots is taken as an indication of lower resilience. Trajectory lengths were quantified as the Euclidean distance between unburned and burned subplot pairs in the two‐dimensional NMDS ordination. Angles were determined using the “trajectoryAngles2D” function in the “ecotraj” package (De Cáceres et al., 2019). The function uses the two‐dimensional NMDS ordination coordinates of unburnt and burnt to determine the angle of change relative to the *y*‐axis (0°).

We acknowledge that our data only capture short‐term changes in composition in the first growing season following fire. Nevertheless, previous studies (e.g., Legg et al., 1992; Maltby et al., 1990) have indicated that effects apparent in the immediate aftermath of fires can persist for extended periods. Thus, whilst our monitoring only makes an initial assessment of vegetation change, it is important for understanding how wildfires affect community structure.

## 3 RESULTS

Burn severity varied greatly within and between individual wildfires (mean pCBI = 2.9, range = 1.0–4.1; mean ground pCBI = 1.1, range = 0.3–1.8; Table 1). Mean pCBI varied 1.6‐fold between wildfires but up to 1.7‐fold within fires. Plots in drier *Calluna*‐dominated communities (National Vegetation Community H12) generally burned at higher severities (mean pCBI = 3.9) than those associated with bog and mire communities (NVC M19, M20, M25a; mean pCBI = 2.3).

### 3.1 Burn severity effects on species richness and diversity

**FIGURE 1:**
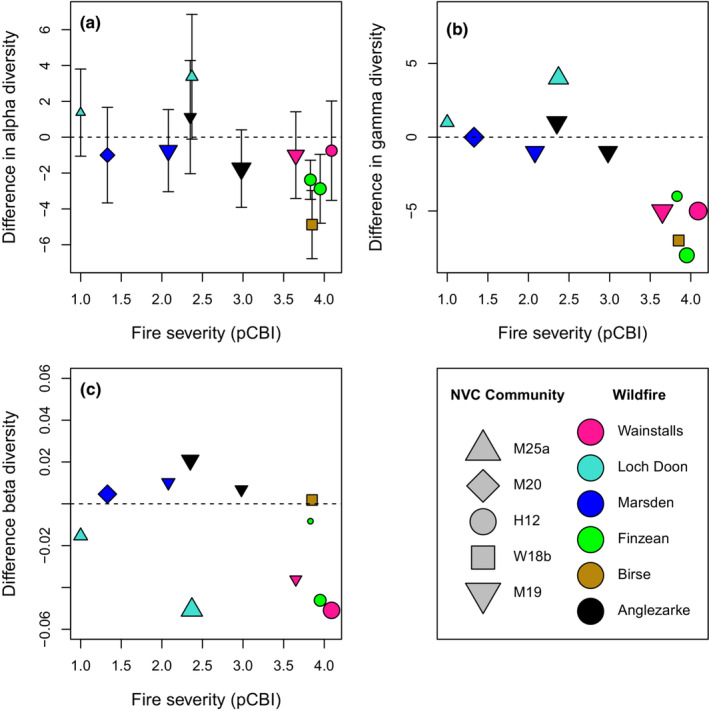
Differences in plant community diversity between burned and unburned subplots as a function of burn severity (pCBI) for six wildfires. Colours demarcate individual fires whilst symbols reflect the National Vegetation Classification community type assigned to each individual monitoring plot. Species presence/absence was recorded in eight quadrats per plot. Results report the (a) mean quadrat‐level species richness (alpha diversity, mean ± SD), (b) the total plot‐level (gamma) diversity (c) and the beta diversity (compositional difference) across quadrats within a plot. Symbol size within each subfigure is proportional to unburned diversity.

**FIGURE 2:**
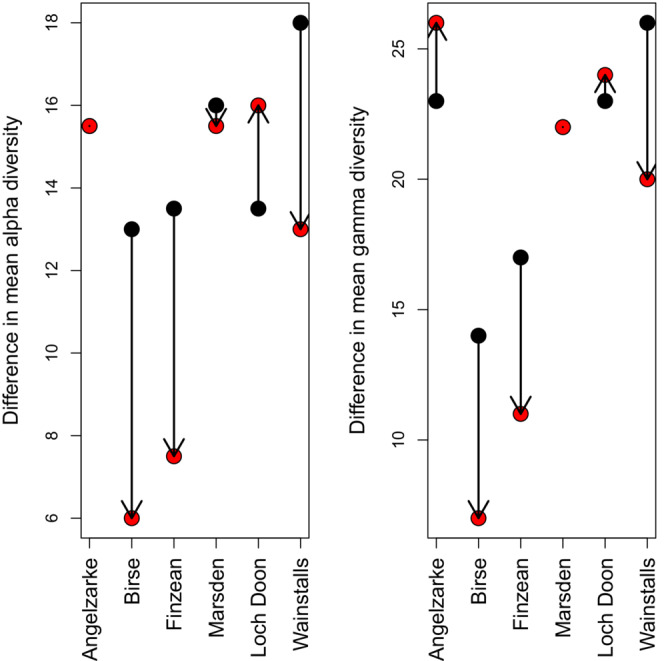
Difference in mean fire‐level alpha (left) and gamma (right) diversity between unburned (black) and burned (red) subplots. Alpha diversity is represented as the mean plot‐level species richness within a fire, gamma diversity is the total number of species recorded for a given fire. Note sampling effort was reduced at the Birse fire (one rather than two paired plots). This will reduce gamma diversity for this site relative to the others but is unlikely to alter the direction of its unburned‐burned subplot trend.

Both mean quadrat‐level species richness and plot‐level gamma diversity were generally reduced following wildfire except in the *Molinia*–*Potentilla* mire community (Loch Doon wildfire) where diversity was higher in burned areas (Figure 1). For both the quadrat and plot scales, declines in richness and diversity were significantly greater at higher burn severities (*r* = −0.60, *p* = .051 and *r* = −0.80, *p* = .003, respectively). The impact of increased burn severity was, however, much greater on plot‐level gamma diversity. There was no evidence of a significant burn severity effect on plot‐level beta diversity (Figure 1; *r* = −0.31, *p* = .331). However, blanket mire communities (i.e., NVC = M19/M20, *Eriophorum* dominated) tended to show slightly increased beta diversity in burned areas. Drier heathland and woodland communities (dwarf shrub‐dominated—NVC = H12, W18b) had lower beta diversity, although they had also burned at the highest severities. There were noticeably different trends in fire‐level mean and total species richness (Figure 2). Fires at sites associated with drier heathland communities (Wainstalls, Finzean) generally saw declines in richness, whilst sites characterized by mire or bog communities tended to be more resilient or even see increases in richness.

### 3.2 Burn severity effects on key plant functional types

**FIGURE 3:**
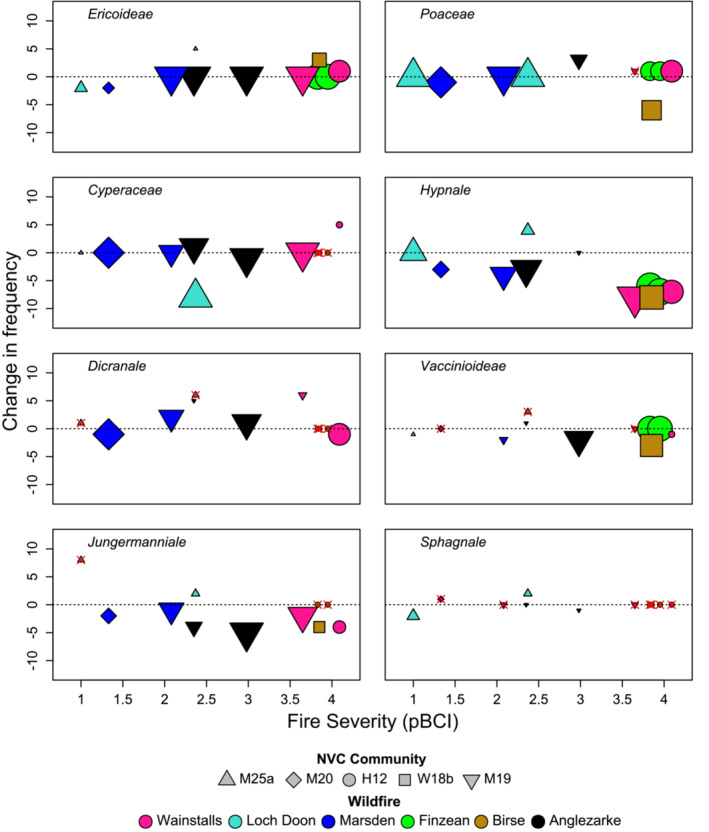
Differences in plant lifeform abundance between burned and unburned paired plots across six different wildfires. Changes in abundance are expressed as the mean difference in frequency of occurrence across eight 1 m^2^ quadrats randomly located within each subplot. Symbol shape and colour, respectively, indicate the pre‐fire plant community and fire location. Symbol size is proportional to the lifeform's pre‐fire abundance. Symbols with a red X through them indicate situations where the lifeform was not recorded in unburned subplots.

Most vascular plant lifeforms were resilient to fire showing generally small changes in frequency following burning and limited evidence of a response to variation in pCBI (Figure 3). Sedges (Cyperaceae) and grasses (Poaceae) appeared to be particularly resilient. Results were inconsistent for the two dwarf shrub groups. Both showed limited differences between burned and unburned areas, but whilst the Ericoideae had a slight tendency to be more frequent at high severities, and less frequent at low severities, the Vacciniodeae had a greater propensity to be somewhat less frequent in burned areas regardless of severity.

In contrast to vascular plants, bryophytes were strongly affected by burning (Figure 3). Liverworts (Jungermanniale) were less frequent in nearly all burnt subplots apart from one location at the lowest fire severity. The frequency of Hypnales (pleurocarpous mosses) was lower in burnt subplots with reductions increasing noticeably as fire severity increased. Dicranales generally showed higher frequency in burnt subplots, though there was limited evidence of a fire severity effect. *Sphagnum* was absent from the majority of plots before the burns, but where it was present, there was no noticeable impact of burning or fire severity.

### 3.3 Plant community resilience in relation to varying burn severity

**FIGURE 4:**
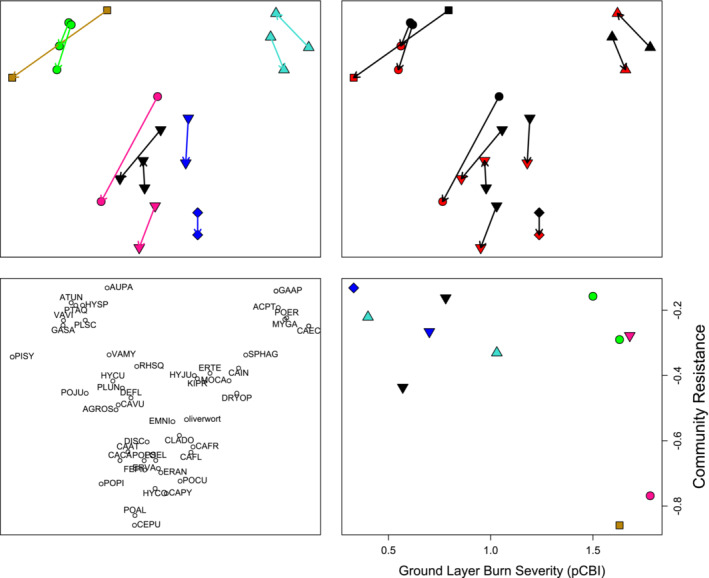
Results of a two‐dimensional Non‐Metric Multidimensional scaling (stress = 0.11) evaluating plant community resilience to wildfire. The individual panes show: (a) compositional differences between burned and unburned subplots as a function of fire identity and NVC community type (symbology as per Figure 1); (b) compositional shifts as a function of NVC community type and status where red = burned; black = unburned; and (c) species centroids (species abbreviations are reported in the Supplementary Material). (d) illustrates the association between burn severity (ground layer pCBI) and community Resilience to fire, which is defined as the Euclidean distance between burned and unburned subplots within the ordination space.

The selected, two‐dimensional NMDS solution had a stress of 0.11. Plots were generally clustered by pre‐fire community type in the resulting ordination (Figure 4). Plot positions on the first axis of the ordination thus represent a moisture and soil/peat depth gradient. Drier heathland and woodland plots (NVC = H12, W18b), to the left of axis one, were strongly associated with *Vaccinium* spp., *Pteridium aquilinum* L., and the pleurocarpous moss *Hylocomium splendens* (Hedw.) Schimp. By contrast, mire communities (NVC = M19, M20, M25a) associated with *M. caerulea*, *Potentilla erecta* (L.) Raeusch., *Myrica gale*, and *Sphagnum* spp. were located on the far right. Mire communities were more centrally located, indicating their intermediate status and association with generalist species such as *C. vulgaris* and *Deschampsia flexusosa* (L.) Trin. Axis 2 of the ordination differentiated between plots with abundant pleurocarpous mosses and dwarf shrubs, and those more strongly associated with sedges, particularly *Eriophorum* spp., and acrocarpous mosses—this axis therefore also differentiated the bog and heath communities (NVC = M19, H12, W18b) from the sedge‐dominated mire communities but related particularly strongly to cryptogam community composition and the structure and function of the moss layer that often underlies vascular vegetation in peatland and heathland habitats.

Wildfire had a relatively consistent effect on vegetation composition as evidenced by the similar trajectories of change angles between unburned and burned subplots (Figure 4, Table 2). With the exception of the bog community plots, burned subplots tended to be more strongly associated with acrocarpous mosses, sedges, and grasses and less associated with pleurocarpous mosses and dwarf shrubs. When examining the relationship between community resilience (i.e., distance separating burned and unburned subplots), the extent of change in community composition was weakly correlated with ground layer fire severity (*r* = 0.53, *p* = .09) as estimated by the pCBI scores assigned to this specific layer. This pattern was particularly apparent for mire and bog community types. Heath (or heathland woodland understory) communities burned at the highest severities but showed variable differences in composition with two plots showing rather little difference and the single woodland understory plot showing the greatest extent of change recorded. It should be noted that dry heathland‐type communities at Birse and Finzean had experienced fire more recently than other sites. However, dropping these sites from the analysis would not meaningfully alter the key patterns observed in relation to changes in diversity and community resilience.

**TABLE 2:** Vegetation trajectory properties associated with the six wildfires sampled.

Wildfire	Trajectory property	
	Length	Angle (°)
Anglezarke	0.30 ± 0.19	292 ± 91
Birse	0.86	242
Finzean	0.22 ± 0.09	206 ± 4
Loch Doon	0.28 ± 0.08	230 ± 144
Marsden	0.20 ± 0.08	182 ± 3
Wainstalls	0.52 ± 0.35	212 ± 6

*Note*: Length was quantified as the Euclidean distance between unburned and burned subplot pairs in the two‐dimensional NMDS ordination. Angles represent the angle of change from unburned to burned plots relative to the *y*‐axis (0°).

## 4 DISCUSSION

The fires we surveyed covered a range of habitat types that broadly describe a hydrological and edaphic gradient from dry heathlands with shallow organic soils to blanket bogs. The fires displayed a range of severities, as detected by the pCBI visual assessment method, and a number of sites included areas showing signs of consumption or charring of the upper few centimeters of peat—this often occurred in isolated “hotspots.” whilst our sampling specifically sought to characterize changes in composition associated with differing levels of severity, none of our fires had extensive very high severity effects documented in some previous studies (e.g., Davies et al., 2013; Maltby et al., 1990). In those cases, significant impacts were evidenced by, for example, large areas of bare eroding peat, exposure of tree roots, significant drifting ash deposits, or widespread exposure of mineral soil. Associations were also found between pCBI scores and moisture codes of the Canadian Fire Weather Index System (Van Wagner, 1987) that are designed to reflect the moisture status of partially decomposed organic matter (F‐layer; duff) and deeper organic soils.

Changes in alpha and gamma diversity at the plot level varied with burn severity and habitat type. Both alpha and gamma diversity declined significantly as burn severity (pCBI) scores increased, though this effect was inevitably somewhat confounded by the pre‐fire vegetation composition—all the plots that burned at high severity were drier heathland or pine forest communities whilst blanket bogs burned less severely. High water tables and deep, saturated layers of bryophytes make bog communities more resilient to burning at high severities and more resilient to the effects of wildfire, at least during periods when water tables are close to the surface. Drier, shallow organic soils found in heathlands and pine woodlands are more likely to ignite and smoulder. Durations of soil surface and subsurface heating have been shown to control the survival of many of the plants, and soil seedbanks, found within peatland and heathland ecosystems (Granström & Schimmel, 1993; Kelly et al., 2016; Lee et al., 2013). Interestingly, changes were more apparent for gamma compared with alpha diversity. This suggests that increases in burn severity may disproportionately affect rarer species that occur in smaller or more isolated/patchy populations. Higher severity fires may also burn less patchily resulting in more consistent reductions in plant populations across the plots and thus substantively reduced gamma diversity. Previous research using experimental fires has suggested that differences in burn severity can significantly increase beta diversity in heathland and peatland plant communities (Grau‐Andrés, Davies, et al., 2019), but such an effect was not detectable for the wildfires we studied.

Impacts on the abundance of various plant functional types were minor, this is perhaps unsurprising given the very long association between fire and heathlands. Such associations are evidenced in both the palaeoecological record (e.g., McCarroll et al., 2017) and in evolutionary responses to fire in heathland plants (Bargmann et al., 2014; Vandvik et al., 2014). There was a weak signal of increased Ericoideae abundance with burn severity, a result seen in previous studies (e.g., Davies et al., 2010). Such responses can be attributed to the ability to resprout following fire, as well as smoke and heat‐stimulated germination. This pattern suggests our fires were not of severities high enough to cause mortality of seeds and rootstocks across substantial areas though we did encounter “hotspots” with very limited regeneration. Where smouldering fires are extensive, mortality of the seedbank and the short dispersal distances of some moorland species (Gilbert & Butt, 2010) may lead to reduced resilience. Tussock‐forming grasses and sedges were also resilient to increased burn severity due to their ability to resprout from insulated basal buds (Grant et al., 1963; Taylor et al., 2001). Among the cryptogam species encountered, acrocarpous mosses tended to show increased abundance irrespective of severity whilst pleurocarpous species tended to decline following fire. The former groups are rapid colonizers of bare peat substrates with traits linked to abundant propagule production and moisture conservation. The latter, by contrast, are generally damaged or consumed during fire. Previous work (Hobbs, 1988) has suggested that liverworts may be sensitive to fire, and we found that such species also declined irrespective of burn severity. Some of these patterns—impacts on cryptogam composition and resilience of tussock‐forming graminoids—are remarkably similar to those seen in studies of severe tundra fires, and indeed, a number of the species involved are the same (Barrett et al., 2012; Bret‐Harte et al., 2013). *Sphagnum* was comparatively rare in our plots and was absent even pre‐fire from the drier communities that burned at the highest severities. This limits our ability to infer the resilience of this key peat‐building species to varying wildfire severity. The lack of *Sphagnum* at our sites may be an indication of past histories of anthropogenic impact in the area including drainage, nutrient deposition, and possibly fire (Davies, 2016).

Previous research in other peatland systems suggests varying support for the importance of interactions between vegetation composition, environmental conditions, and wildfire severity in determining ecosystem resilience to fire. Such effects have been observed in the context of multiple ecosystem functions including carbon dynamics, hydrological function, and primary productivity. Thus, Lecomte et al. (2005) linked lower severity fires to more rapid paludification compared to higher severity burns (Lecomte et al., 2005), and Rocha and Shaver (2011) demonstrated burn severity effects on net ecosystem exchange (Rocha & Shaver, 2011). For hydrological properties, pre‐fire bryophyte species identity has been shown to control post‐fire water repellency (Moore et al., 2017), hydrology, and subsequent moss recolonization (Lukenbach et al., 2016), but Holden et al. (2014) found no differences in near‐surface hydrological parameters between peatlands recently burned by managed versus wildfires. In the boreal forest, variation in burn severity has been linked to differences in aspen versus black spruce regeneration and post‐fire dominance of key plant functional groups (Johnstone & Kasischke, 2005). By contrast, Grau‐Andrés et al. (2017) and Taylor et al. (2017), respectively, showed recovery of *Sphagnum* photosynthetic capacity less than 2 years following fire irrespective of severity and regeneration from growth innovations below the capitulum even after extended heating. Some previous research has suggested that peatlands may be comparatively resilient to occasional wildfires. For instance, Jin et al. (2012) showed recovery of primary productivity of North American boreal forests within 5–8 years; Clarke et al. (2015) concluded Australian alpine bogs could return to unburnt conditions within 20 years; and Kuhry (1994) demonstrated that surface fires in boreal peatlands had minimal effects of vegetation composition but could limit peat accumulation if frequent enough. Nevertheless, other studies have detected ecological legacies from high severity wildfires that have lasted at decadal timescales or longer (e.g., Maltby et al., 1990; Sillasoo et al., 2011; Thomas et al., 1994).

Given these varying responses, in the United Kingdom, we still require information on how bog communities (sensu Averis et al., 2004) respond to high severity fires that result in ignition and widespread consumption and scorching of peat. Whilst we did observe such impacts, our plots generally occurred in smaller isolated “hot spots.” Limited previous research has shown severe wildfires that burn blanket peatlands can produce charred, granulated, and unconsolidated peat surfaces that severely limit seedling recruitment (Legg et al., 1992). Initial recovery in such settings was dominated by acrocarpous mosses, and even 8 years following the fires, <6% of the area was occupied by vascular vegetation (Maltby et al., 1990). Exact trajectories of cryptogamic vegetation development were strongly related to soil surface chemical and physical characteristics, themselves a reflection of the interaction of burn severity and pre‐fire soil type (Thomas et al., 1994). We detected similar patterns in our study where the largest changes in vegetation composition were generally associated with the most severely burned locations and increased abundance of acrocarpous moss species, graminoids, and forbs. The exception to this rule appeared to be the drier heathland site at the higher severity Finzean fire, which was relatively resilient in terms of composition. This is partly ascribable to substantial *Calluna* seedling regeneration and rapid vegetation regeneration of *Pteridium aquilinum*.

In summary, our findings suggest that many peatland species are resilient to the effects of even moderately severe fire. Nevertheless, the magnitude of community change was positively correlated with burn severity. Furthermore, certain plant groups, particularly pleurocarpous mosses and liverworts, are, at least in the short‐term, impacted by fire. We found evidence that increased burn severity may be tied to reductions in plant diversity at multiple spatial scales through some communities showed increased diversity after fire. Such findings require experimental validation given that burn severity and community type were confounded in our data. Our results indicate that the short‐term ecological outcomes of wildfire are a result of complex inter‐relationships between pre‐fire biotic and abiotic conditions, and fire weather conditions that control fuel availability and fire behaviour. Understanding community‐specific responses to wildfire will require wider and, more importantly, longer‐term data. One must also recognize that compositions we witnessed on sites with deep peat soils suggest they were in a degraded condition prior to the wildfires and reference to a pre‐fire condition may be less preferable than to a representative “intact” reference composition. To mitigate the potential for high severity fires with negative consequences for plant diversity and composition, peatland restoration through rewetting remains a priority. Drier sites with shallow (< 50 cm) organic soils may be particularly at risk from higher severity fires in the context of a changing climate, and fuel and fire risk management should be a priority. Future management actions and policies must consider the interaction between fuel availability and climate during peatland restoration to mitigate the potential for extensive severe burns.

## AUTHOR CONTRIBUTIONS

**G. Matt Davies:** Conceptualization (equal); data curation (equal); formal analysis (lead); funding acquisition (equal); investigation (supporting); methodology (equal); project administration (lead); supervision (lead); visualization (equal); writing – original draft (lead). **Alan Gray:** Conceptualization (equal); data curation (equal); formal analysis (supporting); funding acquisition (equal); investigation (supporting); methodology (equal); project administration (supporting); supervision (supporting); writing – review and editing (equal). **Simon C. Power:** Formal analysis (supporting); visualization (supporting); writing – review and editing (supporting). **Rut Domènech:** Data curation (equal); formal analysis (supporting); investigation (lead); methodology (supporting); project administration (supporting); writing – review and editing (equal).

## CONFLICT OF INTEREST STATEMENT

Outwith this study, the authors associated with this paper have received funding or in‐kind support from the following organisations: Scottish Natural Heritage, the Royal Society for the Protection of Birds, the Game and Wildlife Conservation Trust, the Heather Trust, Natural England, the National Trust for Scotland, as well as private and public landowners and managers.

## Supporting information


**Data S1:** Supporting Information

## Data Availability

All relevant data associated with this paper are available in the Supporting Information. Additionally, both the data and R scripts are archived with OSF (DOI: https://doi.org/10.17605/OSF.IO/R463E).
